# Localized Gastrointestinal Light Chain (AL) Amyloidosis Under Surveillance for Five Years: A Case Report

**DOI:** 10.1002/deo2.70343

**Published:** 2026-05-06

**Authors:** Shunsuke Kojimahara, Keiichi Tominaga, Akira Yamamiya, Mimari Kanazawa, Takanao Tanaka, Takeshi Sugaya, Nagaaki Katoh, Atsushi Irisawa

**Affiliations:** ^1^ Department of Gastroenterology Dokkyo Medical University School of Medicine Tochigi Japan; ^2^ Department of Medicine (Neurology and Rheumatology) Shinshu University School of Medicine Nagano Japan

**Keywords:** AL amyloidosis, light chain amyloidosis, localized gastrointestinal amyloidosis, small‐bowel endoscopy, surveillance

## Abstract

Amyloidosis, characterized by the deposition of abnormal protein fibrils in organs, is classified as systemic or localized. Amyloid light chain (AL)‐type localized amyloidosis is uncommon, particularly when confined to the gastrointestinal tract. Herein, we report a case of localized gastrointestinal AL‐type amyloidosis that was incidentally detected and remained endoscopically and clinically stable over a 5‐year follow‐up period.

The patient, a man in his 40s, had undergone a colonoscopy for colorectal cancer screening, during which scattered erosions were detected in the colon. Amyloid deposits were identified on biopsy. No cardiac or renal involvement, or evidence of multiple myeloma, was found. He was ultimately diagnosed with localized AL‐type gastrointestinal amyloidosis. Following diagnosis, the patient underwent regular surveillance with transoral and transanal small‐bowel endoscopy. No endoscopic progression or gastrointestinal symptoms were observed during the follow‐up period, and the patient remained asymptomatic. This case suggests that conservative observation may be reasonable in carefully selected patients after exclusion of systemic involvement.

## 1 Introduction

Amyloidosis, defined as the deposition of abnormal protein fibrils (termed amyloids) in organs, is clinically categorized as either systemic or localized. Systemic amyloidosis causes damage to multiple organs, such as the kidneys, heart, liver, and digestive tract, and often has a poor prognosis [1]. Systemic gastrointestinal amyloidosis is commonly diagnosed in association with multiple myeloma and presents with diverse symptoms, including diarrhea, gastrointestinal bleeding, malabsorption, and obstruction due to peristalsis failure [2]. In comparison, amyloid light chain (AL)‐type localized amyloidosis derived from immunoglobulin light chains is relatively rare; cases confined to the gastrointestinal tract are exceedingly rare, and reported cases remain limited [1, 2].

In this report, we describe a case of localized gastrointestinal AL‐type amyloidosis that was incidentally discovered and subsequently confirmed to have remained endoscopically and clinically stable over 5 years of surveillance.

## 2 Case Report

The patient, a man in his 40s with no significant past medical history, was referred to our hospital to undergo further evaluation after a colonoscopy performed due to a positive fecal immunochemical test revealed scattered erosions with amyloid deposits. No abnormalities in vital signs, abdominal examination, or other physical findings were noted. Laboratory results on admission are presented in Table 1. The laboratory data were as follows: aspartate aminotransferase, 36 U/L; alanine aminotransferase, 28 U/L; alkaline phosphatase, 191 U/L; lactate dehydrogenase, 195 U/L; C‐reactive protein, 0.01 mg/dL; prothrombin time activity, 115 %; activated partial thromboplastin time, 28.5 s; carcinoembryonic antigen, 1.2 ng/mL; and carbohydrate antigen 19‐9 5, U/mL, revealing no significant abnormalities in liver function, inflammatory markers, or tumor markers. Amyloid A protein was mildly elevated at 3.5 mg/L. Serum free light chain levels were within the normal range, with free κ, free λ, and κ/λ ratio of 9.5 mg/L, 15.4 mg/L, and 0.62, respectively. Serum immunofixation electrophoresis revealed no monoclonal protein. Urinalysis was negative for proteinuria, occult blood, and Bence–Jones protein. Furthermore, bone marrow biopsy showed no significant pathological abnormalities, such as abnormal plasma cell proliferation or amyloid deposition. Cardiac echocardiography revealed no diffuse myocardial hypertrophy, and the electrocardiogram showed a normal sinus rhythm. Contrast‐enhanced computed tomography (CT) of the head, chest, and abdomen demonstrated no abnormalities.

**TABLE 1:** Laboratory results on admission.

AST	36	U/L	WBC	6.20	×10^3^/µL
ALT	28	U/L	RBC	4.47	×10^6^/µL
ALP	191	U/L	Hb	14.2	g/dL
LDH	195	U/L	Plt	191	×10^9^/L
γGTP	18	U/L	Ht	42.5	%
T‐Bil	0.7	mg/dL	PT%	115	%
D‐Bil	0.2	mg/dL	APTT	28.5	sec
TP	6.5	g/dL	HBsAg	0.00	IU/mL
Na	133	mmol/L	HCVAb	Negative	
K	3.8	mmol/L	Anti‐HIV antibody	Negative	
Cl	90	mmol/L	T‐SPOT	Negative	
BUN	18	mg/dL	RF	5	IU/mL
Cre	0.81	mg/dL	Complement (CH50)	46	U/mL
CRP	0.01	mg/dL	ANA	×20	
IgG	856	mg/dL	BNP	6.7	pg/mL
IgM	95	mg/dL	Troponin T	0.006	ng/mL
IgA	120	mg/dL	CEA	1.2	ng/mL
			CA19‐9	5	U/mL
			Ferritin	129.6	ng/mL
			Serum amyloid A protein	3.5	mg/L

**FIGURE 1:**
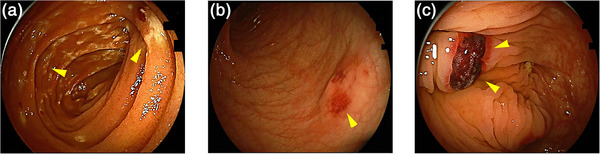
Initial findings of small‐bowel endoscopy at diagnosis. (a) Transoral small‐bowel endoscopy showing multiple small whitish elevations (arrowhead) extending from the duodenum to the jejunum. (b) Transanal small‐bowel endoscopy showing scattered erosions (arrowhead). (c) Transanal small‐bowel endoscopy showing hematoma formation at the biopsy site (arrowhead).

**FIGURE 2:**
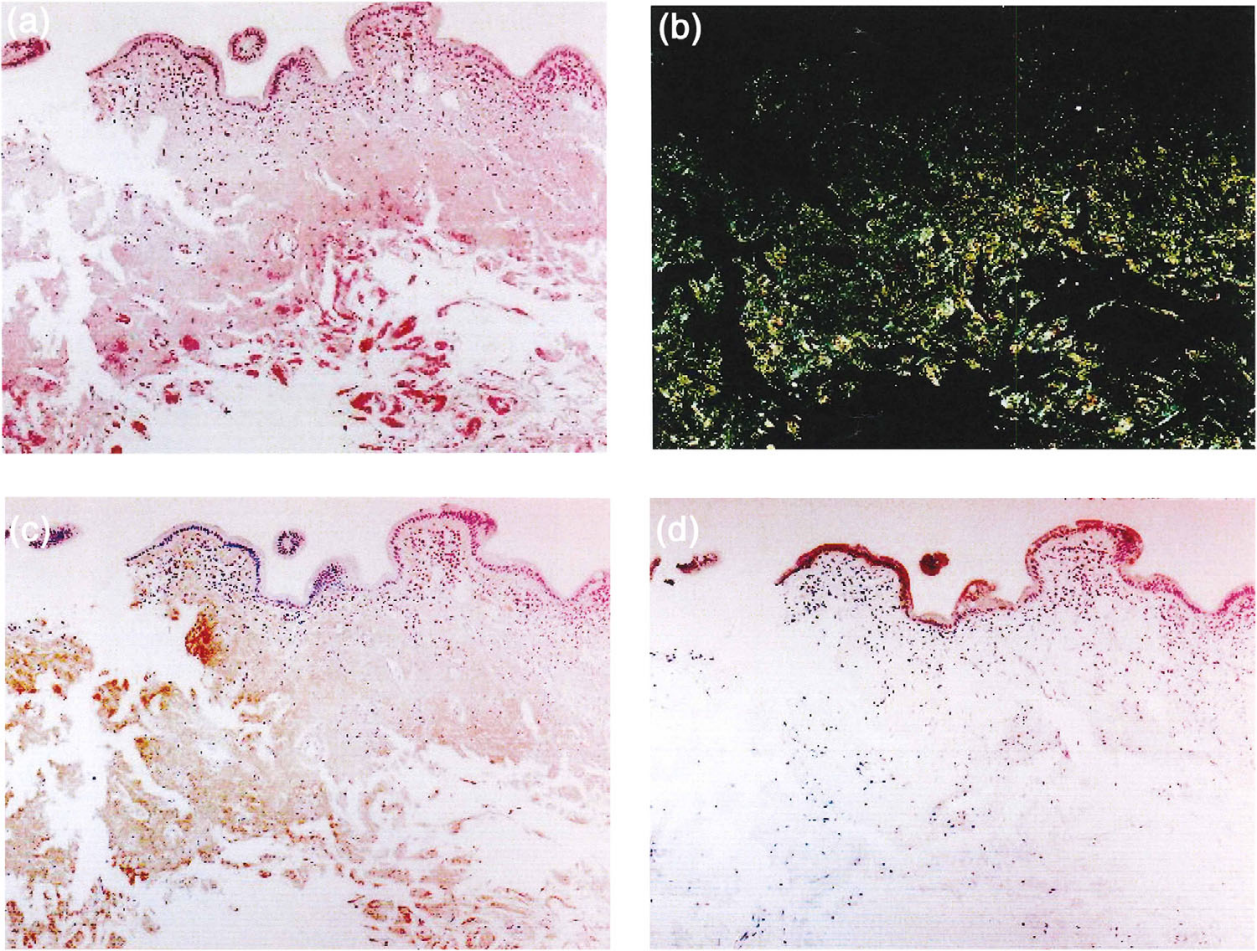
Histopathological findings at diagnosis using biopsy specimens obtained from the duodenum. (a) Congo red staining of the duodenal biopsy (original magnification ×40). (b) Congo red staining under polarized light (original magnification ×40). (c) Immunohistochemistry for anti‐κ antibody: positive (original magnification ×40). (d) Immunohistochemistry for anti‐λ antibody: negative (original magnification ×40).

Transoral small‐bowel endoscopy revealed multiple small, whitish elevations extending from the duodenum to the jejunum (Figure 1a). Transanal small‐bowel endoscopy revealed scattered erosions in the colon (Figure 1b,c). Congo red staining of biopsy specimens obtained from the duodenum revealed amyloid deposits. Immunohistochemical staining was positive for anti‐κ antibodies and negative for anti‐λ, anti‐transthyretin, anti‐AA, and anti‐Aβ_2_M antibodies (Figure 2a–d). Comprehensive systemic evaluation revealed no evidence of systemic amyloidosis. Accordingly, the patient was diagnosed with AL κ‐type amyloidosis based on immunohistochemistry. As no amyloid deposits were detected in other organs, a classification of localized AL‐type gastrointestinal amyloidosis was made.

**FIGURE 3:**
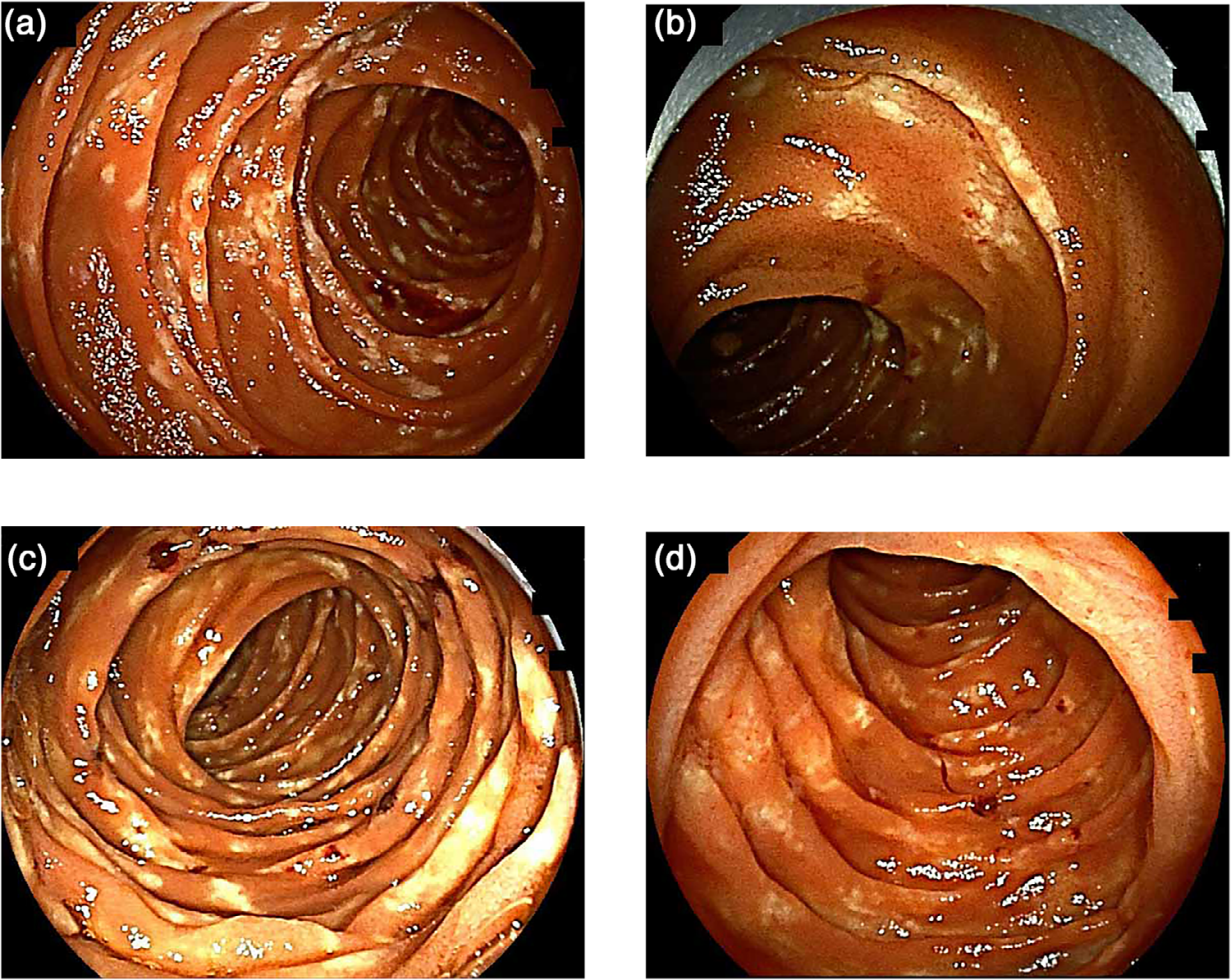
Small‐bowel endoscopic findings during 5‐year surveillance. (a) Year 1: Transoral endoscopy showing multiple small whitish elevations extending from the duodenum to the jejunum. (b) Year 2: Transoral small‐bowel endoscopy. (c) Year 4: Transoral small‐bowel endoscopy. (d) Year 5: Transoral small‐bowel endoscopy. The whitish amyloid deposition remained largely unchanged throughout the follow‐up period.

After diagnosis, the patient underwent regular surveillance with small‐bowel endoscopy using both transoral and transanal approaches. The surveillance schedule was as follows: Year 1: transoral; Year 2: transoral; Year 3: transanal; Year 4: transoral; Year 5: combined transoral and transanal examinations (Figure 3a–d and Figures S1 and S2). Over the 5‐year surveillance period, no progression or notable changes in the endoscopic findings were observed. In addition, no gastrointestinal symptoms, including hematochezia, persistent diarrhea, or impaired motility, developed. Thus far, the patient has remained completely asymptomatic, with a stable clinical course.

## 3 Discussion

Herein, we present a case of localized AL‐type amyloidosis of the gastrointestinal tract incidentally discovered following a positive fecal immunochemical test in an otherwise asymptomatic patient. No progression in symptoms or endoscopic findings has been observed throughout 5 years of surveillance.

Cowan et al. reported in a single‐center series that none of the 16 investigated patients with localized gastrointestinal amyloidosis progressed to systemic disease during follow‐up, and all demonstrated favorable survival outcomes [3]. Recent systematic reviews have similarly shown that cases with localized gastrointestinal involvement are typically managed with supportive care and close observation [4]. In addition, several reports have described asymptomatic cases of localized intestinal AL amyloidosis with similarly indolent clinical courses. Kuroha et al. reported three asymptomatic patients detected following positive fecal immunochemical tests, all of whom remained stable without treatment over long‐term follow‐up [5]. Furthermore, Alshehri et al. emphasized that localized amyloidosis represents a site‐restricted plasma cell disorder with a minimal risk of progression to systemic disease [6]. The present case is consistent with these previous observations, as no clinical or endoscopic progression was observed over a 5‐year follow‐up period. Notably, the current case is distinguished by detailed longitudinal endoscopic surveillance, which allowed confirmation not only of clinical stability but also of sustained endoscopic quiescence. Taken together with previous reports, these findings suggest that, in carefully selected patients with strictly excluded systemic involvement, no associated complications, and stable endoscopic findings, conservative management with close observation may represent an appropriate strategy.

Asymptomatic and incidentally detected gastrointestinal amyloidosis are comparatively uncommon. As such, colonoscopy plays a crucial diagnostic role when prompted by diagnostic triggers, such as a positive fecal immunochemical test or the identification of abnormal findings during health screening [2]. Endoscopic features of gastrointestinal amyloidosis include white papules, erosions, ulcers, mucosal fragility, and villous hypertrophy [3, 7]. However, these findings are nonspecific and require distinction from conditions such as inflammatory bowel disease, ischemic colitis, and malignant lymphoma. Consequently, confirmation of apple‐green birefringence under polarized light on Congo red staining of biopsy specimens is essential for a definitive diagnosis, followed by immunostaining or mass spectrometry to identify the amyloid protein type [8]. In particular, mass spectrometry‐based subtyping has recently arisen as the gold standard, addressing the limitations of immunostaining [7]. Although mass spectrometry was not performed, AL κ‐type amyloidosis was diagnosed based on immunohistochemistry in this case. As mass spectrometry‐based proteomic analysis is currently considered the gold standard for amyloid subtyping, the present diagnosis should be interpreted with caution. Comprehensive systemic evaluation, including bone marrow biopsy, excluded systemic involvement, thereby supporting a diagnosis of localized gastrointestinal amyloidosis.

Bleeding from gastrointestinal amyloidosis is one of the most common complications and presents a wide clinical spectrum, ranging from chronic iron‐deficiency anemia caused by occult loss to episodes of massive hemorrhage. Biopsy is indispensable for the diagnosis of amyloidosis; however, the mucosa at sites of amyloid deposition is extremely fragile. In AL amyloidosis, deposition of amyloid protein within the small vessel walls of the gastrointestinal tract leads to disruption of vascular architecture and impaired vasoreactivity. Such vascular involvement increases mucosal fragility and predisposes patients to bleeding, either spontaneously or following minor trauma such as endoscopic manipulation. Consequently, procedure‐related complications, including bleeding, hematoma formation, and perforation, may occur, albeit infrequently [9, 10]. The endoscopic findings in this case show a typical submucosal hematoma and erosion in AL amyloidosis. As such, biopsies should be limited to the minimum number required to establish a diagnosis, while meticulous hemostasis and careful post‐procedural observation are essential. Given these considerations, clinicians must approach diagnostic and therapeutic procedures with a high degree of caution.

Localized gastrointestinal AL‐type amyloidosis has been reported in several studies and typically demonstrates a long‐term, asymptomatic, and stable clinical course. This case suggests that conservative observation may be reasonable in carefully selected patients after exclusion of systemic involvement. Continued accumulation of well‐documented cases is expected to refine optimal management strategies, including appropriate follow‐up intervals and indications for intervention.

## Author Contributions

**Shunsuke Kojimahara**: conceptualization. **Keiichi Tominaga**: conceptualization and writing – review and editing. **Mimari Kanazawa**: visualization and writing – original draft. **Akira Yamamiya**: conceptualization and writing – review and editing. **Takeshi Sugaya**: writing – original draft and visualization. **Takanao Tanaka**: visualization and writing – original draft. **Atsushi Irisawa**: writing – review and editing and supervision. **Nagaaki Katoh**: writing – original draft, visualization.

## Conflicts of Interest

The authors declare no conflicts of interest.

## Funding

The authors have nothing to report.

## Ethics Statement

IRB approval was not required.

## Consent

Written informed consent has been obtained from the patient to publish this paper.

## Supporting information




**FIGURE S1** Biopsy‐induced hematoma observed during transanal small‐bowel endoscopy at Year 3.

**FIGURE S2** Transanal small‐bowel endoscopic findings at Year 5. Scattered erosions observed in the colon.

## Data Availability

The authors have nothing to report.
