# Supplementary figures and images for: Localized Gastrointestinal Light Chain (AL) Amyloidosis Under Surveillance for Five Years: A Case Report
Source: DEN Open. 2026 May 6;7:e70343. doi: 10.1002/deo2.70343 (PMC13150062; doi:10.1002/deo2.70343)

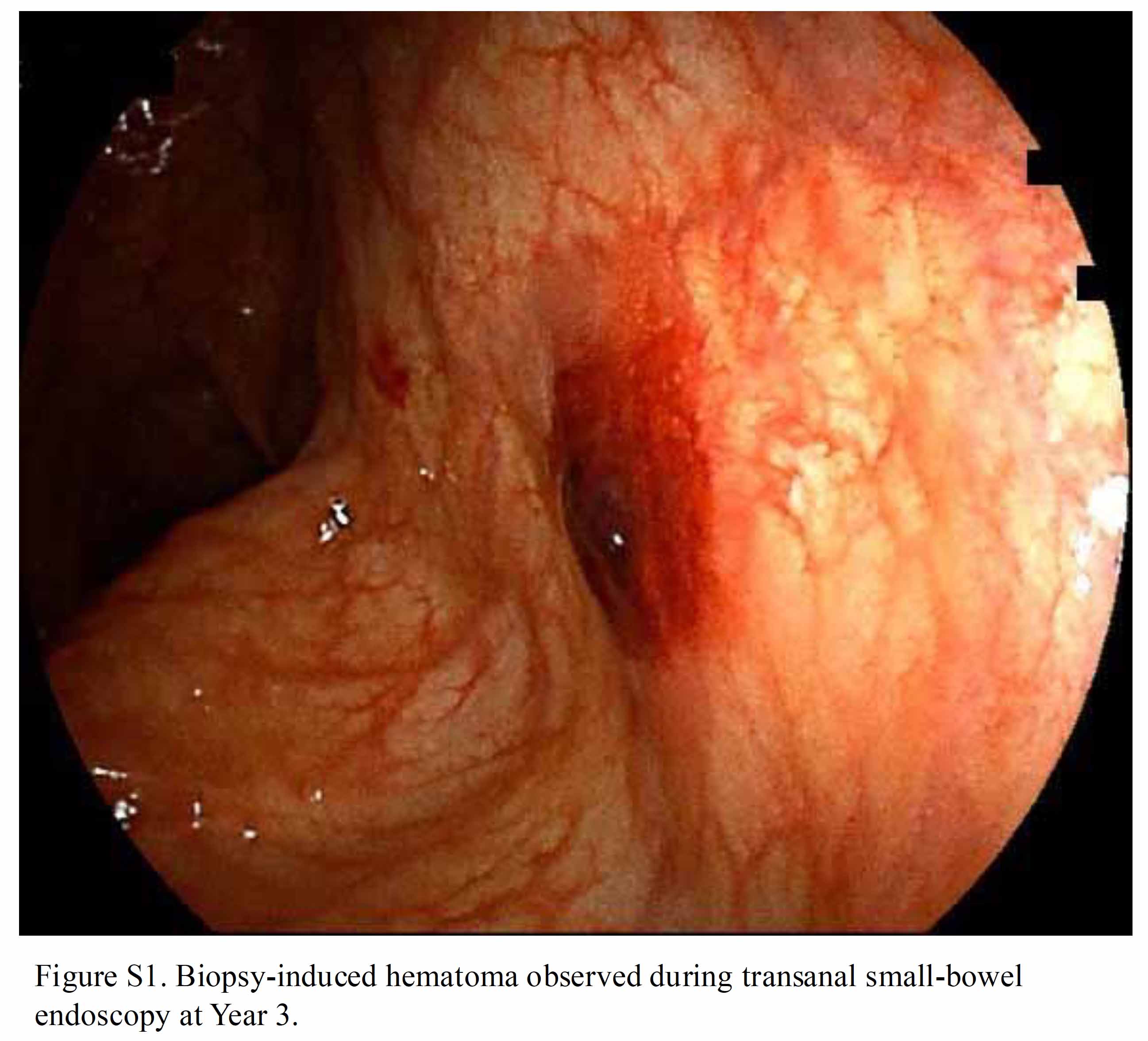

Supplement: Supplementary file 1 — FIGURE S1 Biopsy‐induced hematoma observed during transanal small‐bowel endoscopy at Year 3. [file DEO2-7-e70343-s002.jpg]

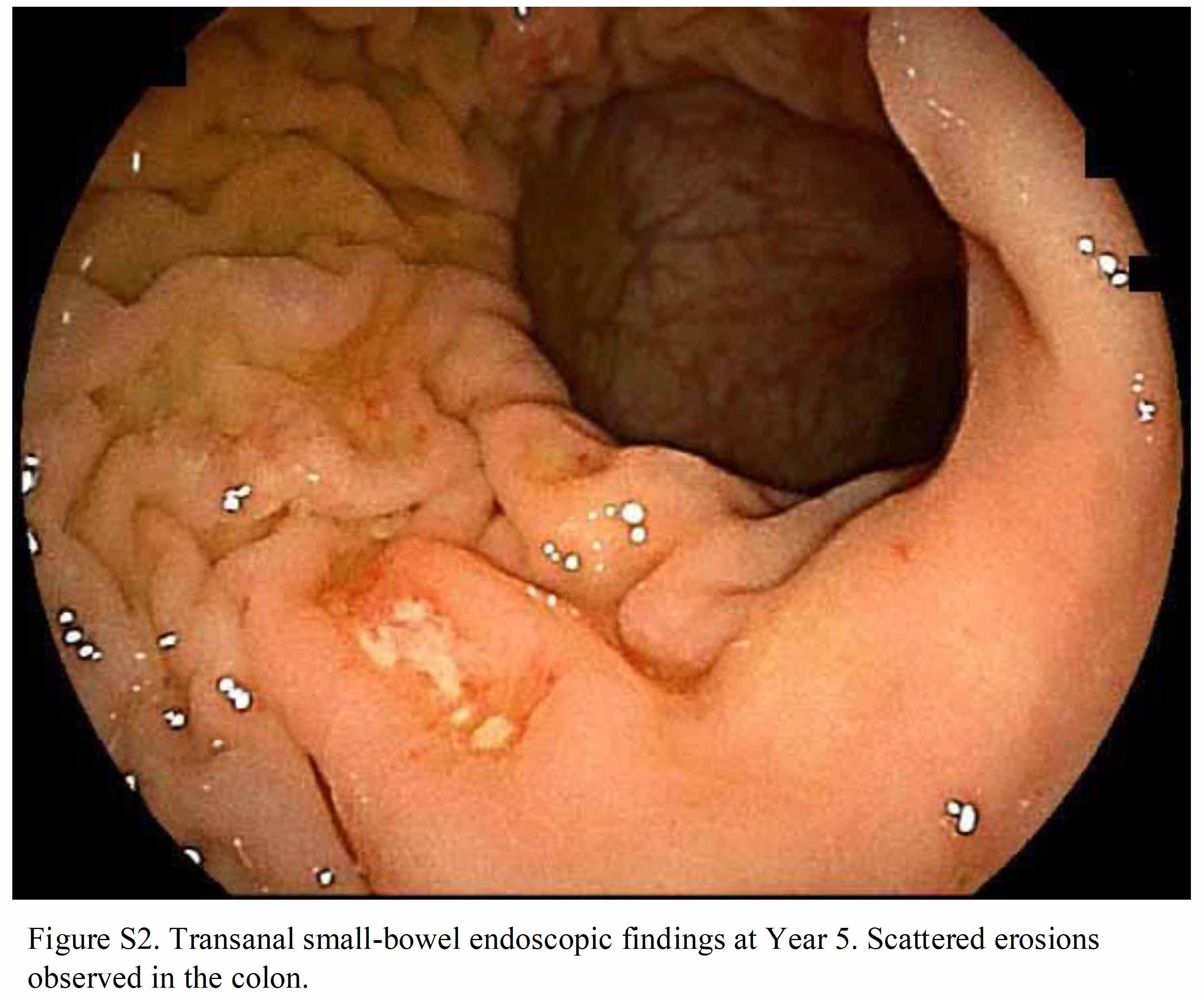

Supplement: Supplementary file 2 — FIGURE S2 Transanal small‐bowel endoscopic findings at Year 5. Scattered erosions observed in the colon. [file DEO2-7-e70343-s001.jpg]
